# Prognostic value of plasma biomarkers for informing clinical trial design in mild-to-moderate Alzheimer’s disease

**DOI:** 10.1186/s13195-025-01745-3

**Published:** 2025-05-02

**Authors:** Yuqi Qiu, Diane M. Jacobs, Karen Messer, David P. Salmon, Cheryl L. Wellington, Sophie Stukas, Carolyn Revta, James B. Brewer, Gabriel C. Léger, Brianna Askew, Lia Donahue, Stephen Kaplita, Vladimir Coric, Irfan A. Qureshi, Howard H. Feldman, The Alzheimer’s Disease Cooperative Study T2 Protect AD Study Group, Gabriel C. Léger, Thomas Obisesan, Amanda Smith, Judith Heidebrink, Jacobo Mintzer, Thomas Ala, Aimee Pierce, Lon Schneider, Judith Heidebrink, Karen Bell, Milap Nowrangi, Amanda Smith, Gregory Jicha, Oscar Lopez, Anton Porsteinsson, Angela Jefferson, Martin Farlow, Christopher van Dyck, Ian Grant, Alan Siegal, Bryan Spann, Alireza Atri, Alan Lerner, Douglas Scharre, Del D. Miller, Elaine Peskind, Jonathan Drake, Aaron Ritter, Charles Bernick, Ralph Richter, Michael Karathanos, Jacobo Mintzer, Olga Brawman-Mintzer, Bernard Baumel, Jeffrey Keller, Andrea Bozoki, Christos Sidiropoulos, Sharon Brangman, Olga Tchikindas, Kiran Bath, David Weisman, Clifford Singer, Maya Lichtenstein, Mary Sano, James J. Peters, Alexander Beyzer, Richard Shubin, Peter McCallister, Stephen Flitman, Cherian Verghese, Sudha Seshadri, Anna Burke, Jeffrey Ross, Mark Brody

**Affiliations:** 1 KLATASDS-MOE, School of Statistics, East China Normal University, Shanghai, China; 2 Division of Biostatistics and Bioinformatics, Herbert Wertheim School of Public Health, University of California San Diego, La Jolla, CA USA; 3 Department of Neurosciences, School of Medicine, University of California San Diego, La Jolla, CA USA; 4 Alzheimer’s Disease Cooperative Study, University of California San Diego, La Jolla, CA USA; 5 Department of Pathology and Laboratory Medicine, Djavad Mowafaghian Centre for Brain Health, University of British Columbia, Vancouver, BC Canada; 6 International Collaboration on Repair Discoveries, University of British Columbia, Vancouver, BC Canada; 7 Biohaven Pharmaceuticals, Inc, New Haven, CT USA

## Abstract

**Background:**

Emerging evidence supports the diagnostic and prognostic utility of plasma biomarkers in Alzheimer’s disease (AD), particularly in early disease stages. We sought to extend these findings by evaluating the prognostic value of plasma biomarkers in a clinical trial of mild-to-moderate AD.

**Methods:**

Post-hoc analyses investigated whether baseline concentrations of plasma biomarkers (Aβ42/Aβ40, T-tau, P-tau181, NfL, and GFAP) predicted change in ADAS-Cog11, CDR-SB, and volumetric MRI among participants in T2 Protect AD, a negative 48-week, phase-2, placebo-controlled trial of troriluzole in mild-to-moderate AD. All trial participants met diagnostic criteria for probable AD. Baseline concentrations of, and 48-week changes in, plasma biomarkers were assessed for association with 48-week change in outcomes using linear regression. Combinations of baseline biomarkers that best predicted change on the ADAS-Cog11 and CDR-SB were identified using least absolute shrinkage and selection operator (LASSO) regression. Biomarker-informed sample size calculations were modeled.

**Results:**

Of 350 trial participants, 319 had all requisite biomarker and clinical outcome data for inclusion in these analyses (mean age 71.5, SD = 8.03; 58.6% female). Higher plasma NfL at baseline predicted worsening scores on the ADAS-Cog11 (effect size (ES) = 1.42, 95%CI = [0.43, 2.41], *p* = 0.026) and CDR-SB (ES = 0.42, 95%CI = [0.10, 0.73], *p* = 0.048). LASSO regression revealed that worsening on the ADAS-Cog11 was best predicted by the combination of baseline plasma NfL, T-tau, and Aβ42/40 ratio, whereas baseline NfL alone best predicted worsening on CDR-SB. Higher baseline NfL predicted increasing ventricular volume (ES = 1.30cm^3^, 95%CI = [0.43, 2.17], *p* = 0.018) and decreasing mid-temporal cortical volume (ES = -0.47, 95%CI = [-0.74, -0.20], *p* = 0.003). Increasing NfL over the 48-week trial was associated with worsening on CDR-SB but not ADAS-Cog11. Modeling of biomarker-informed power calculations revealed that including high NfL as a trial entry criterion could substantially reduce requisite trial sample size.

**Conclusions:**

Elevated baseline plasma NfL predicted more rapid clinical decline and MRI volume loss. Furthermore, increasing plasma NfL concentration over time was associated with worsening on the CDR-SB. Plasma NfL is an easily accessible biomarker that may enhance the design of clinical trials in mild-to-moderate AD.

**Trial registration:**

The T2 Protect AD trial was registered as NCT03605667 on clinicaltrials.gov on 2018-07-27.

**Supplementary Information:**

The online version contains supplementary material available at 10.1186/s13195-025-01745-3.

## Introduction

Advances in the development of ultrasensitive assays now allow concentrations of brain proteins associated with Alzheimer’s disease (AD) pathology, neuronal degeneration, and neuroinflammation to be measured in blood [1, 2]. In addition to confirming the presence of AD pathological changes prior to trial enrollment, informative and validated blood-based biomarkers have potential to enhance AD trial design. For example, they could be used to enrich inclusion criteria, evaluate target engagement, or monitor treatment response. With further development, they may also have utility as sensitive surrogate endpoints in early phase screening trials of therapeutic agents.

There has been a spate of recent research exploring the utility of blood-based biomarkers in the preclinical stages of AD, when disease modifying treatments targeting upstream pathology, such as amyloid beta (Aβ), are most likely to be efficacious [3–6]. In this preclinical population, plasma markers of phosphorylated tau have demonstrated sensitivity to detect changes in the earliest stages of disease and predict cognitive decline [7, 8]. Although the prognostic value of plasma biomarkers in patients with symptomatic AD has been somewhat less well studied, several investigations suggest that plasma neurofilament light (NfL), a marker of axonal damage, may hold particular promise for predicting and tracking changes in patients with mild cognitive impairment (MCI) or dementia due to AD [9, 10].

Here, in a series of post-hoc analyses of data from a phase 2, randomized, placebo-controlled trial of mild-to-moderate AD (RCT; NCT03605667), we examined whether concentrations of plasma biomarkers (Aβ42/40 ratio, T-tau, P-tau181, NfL, and glial fibrillary acidic protein (GFAP)) at baseline predicted change in clinical, and neuroimaging outcomes. We additionally examined the associations between biomarker change over the course of the trial with changes in clinical outcomes. Finally, we explored the potential utility of these plasma biomarkers for informing AD clinical trial design.

## Methods

### Study design and participants

Data were from T2 Protect AD, a double-blind, placebo-controlled, parallel group RCT of troriluzole (BHV-4157), a novel glutamate modulator and prodrug of riluzole, in mild-to-moderate AD. The trial was conducted between July 2018 and December 2021 at 44 US clinical and academic centers under the coordination of the Alzheimer's Disease Cooperative Study (ADCS) and sponsorship of Biohaven Pharmaceuticals, Inc. Details of the trial design and primary results are presented elsewhere [11, 12]. Briefly, participants were 50 to 85 years of age; ambulatory; clinically diagnosed with probable AD by NIA/Alzheimer’s Association guidelines [13]; Mini Mental State Exam (MMSE) score of 14 to 24; modified Hachinski Ischemic Scale score less than 5; MRI scan consistent with a diagnosis of probable AD [13]; on a stable dose of acetylcholinesterase inhibitors and/or memantine; and had an available study partner. Confirmation of AD biomarkers was not a requisite inclusion criterion; nevertheless, of 50 participants who completed an optional lumbar puncture at baseline, 47 (94%) were CSF Aβ42/40 positive, suggesting high clinical diagnostic accuracy among T2 Protect AD participants. Key exclusion criteria were hepatic impairment; other neurodegenerative diseases and causes of dementia; major depressive episode within the six months preceding screening; current serious or unstable medical illness; and MRI evidence of infection, tumor, cortical infarction, extensive white matter disease (Fazekas score > 2), or multiple lacunes in prefrontal or critical memory regions. A total of 350 participants were randomized with 172 assigned to placebo and 178 assigned to treatment with troriluzole. The modified intention to treat (mITT) population included 335 randomized participants who took at least one dose of study medication (i.e., troriluzole or placebo), had a baseline assessment with at least one of the two co-primary clinical endpoints, and had at least one post-baseline efficacy evaluation during the double-blind phase of the trial. The current analysis sample includes 319 participants from the mITT population who had plasma biomarkers measured at baseline (Supplemental Fig. 1).

### Clinical outcome measures

The co-primary clinical outcome measures in T2 Protect AD were 48-week change on the 11-item version of the Alzheimer’s Disease Assessment Scale–Cognitive Subscale (ADAS-Cog11) [14] and change on the Clinical Dementia Rating (CDR) [15] sum of box score (CDR-SB). Both are widely used clinical outcome measures in AD clinical trials. The ADAS-Cog11 is a structured scale that evaluates memory, receptive and expressive language, orientation, ideational praxis, and constructional praxis. Scores can range from 0 to 70 with a higher total score indicating worse cognition. The CDR-SB is a composite rating of cognition and everyday function which incorporates both informant input and direct assessment of performance. Scores on the CDR-SB can range from 0 to 18 with higher scores indicating greater impairment. CDR raters were blind to scores on the ADAS-Cog11 and other cognitive assessments administered in the trial. CDR raters and cognitive assessment raters were blind to participant plasma biomarker concentrations, MRI measurements, and treatment arm assignment. The CDR and ADAS-Cog11 were collected at baseline and weeks 12, 24, 36, and 48.

### Plasma biomarkers

Plasma was collected for biomarker analyses at baseline and week 48. Blood was drawn from a forearm vein into EDTA vacutainer tubes and centrifuged at 2000 × *g* for 10 min at 4 °C in a tabletop centrifuge within 30 min of collection. Plasma was then separated and aliquoted into 2.0 ml polypropylene cryotubes in 1.5 mL fractions and snap frozen within two hours of collection. Samples were stored at the collection site at −80 °C until overnight shipped on dry ice to the National Centralized Repository for Alzheimer's Disease and Related Dementias (NCRAD) which served as the central biobank. Once all collection had been completed, an aliquot of each sample was overnight shipped on dry ice from NCRAD to the laboratory of Dr. Cheryl Wellington at the University of British Columbia where all plasma biomarker analyses were performed. All plasma biomarker analyses were completed between March-June 2021.

All lab personnel conducting biomarker assays were blinded to participant characteristics, clinical outcomes, MRI measurements, and treatment arm. Plasma biomarkers were measured using single molecule array (Simoa) assays (Quanterix, Inc) for beta-amyloid 1–40 (Aβ40), Aβ 1–42 (Aβ42), T-tau (Neurology 3-Plex A Advantage Kit #101995), P-tau181 (P-tau181 V2 Advantage Kit #103714), neurofilament light (NfL; Advantage Kit #102258), and glial fibrillary acidic protein (GFAP; GFAP Discovery Kit #102336). Assays were performed using the HD-X Analyzer following manufacturer’s instructions; each assay run contained an 8-point calibrator curve, two internal kit controls, three plasma controls and up to 83 participant specimens analyzed in a randomized order. Analyses were performed in duplicate and the mean value was reported as the result. For Aβ40, the plasma samples that were > upper limit of quantification (*n* = 5) or > upper limit of detection (*n* = 3) were re-analyzed using a 10 and 25-fold dilution; all other analytes fell within the limits of assay quantification. The average intra-well coefficient of variability (CV) and absolute error were: 4.0–6.0% and 2.7–6.0% for the calibrators and 4.6–6.0% and 6.5–12% for the kit controls, respectively. The inter-plate CV, calculated using the 3 plasma controls included on every run, was 7.9–16%. The average intra-plate CV, calculated using participant specimens, was 3.4–7.4%. Quality assurance and quality control specifications for each analyte are provided in Supplemental Table1.

### Magnetic Resonance Imaging (MRI) measurement

Cranial MRI scans were obtained at screening, week 24, and week 48. All scans were conducted on 1.5 or 3 T scanners using the following protocol: 3-plane localizer; calibration scan (if applicable); sagittal 3D MPRAGE/IRSPGR; axial T2 FLAIR; axial diffusion weighted scan; and axial GRE. Imaging across time points for each participant was conducted using the same scanner and acquisition parameters. Scans were processed and segmented using NeuroQuant software (CorTechs Labs, Inc.). Volumetric measurements (adjusted for intracranial volume) were obtained for hippocampus, entorhinal cortex, precuneus cortex, isthmus of cingulate gyrus, middle-temporal gyrus, supramarginal gyrus, whole brain, and lateral ventricles. Change in these MRI volumes over 48 weeks was assessed using quantitative anatomic regional change (QUARC), a nonlinear serial MRI registration method that enables precise quantification of anatomical changes, including in small regions of interest [16]. The percentage of deformation between screening and week 48 in each of the eight specified regions was quantified.

### Statistical analysis

Because the troriluzole and placebo groups in the T2 Protect AD trial did not differ significantly in participant characteristics (age, education, APOE ε4 carrier status (positive/negative), sex, race) or clinical outcomes (ADAS-Cog11, CDR-SB, MMSE, or MoCA) at baseline, and no statistically significant treatment effect was observed on the ADAS-Cog or CDR-SB [11, 12], treatment arms were combined for these post-hoc biomarker analyses. Nevertheless, treatment arm was included as a covariate in the analyses reported herein to control for potential randomization-induced variance and to maintain the integrity of the experimental design inherent to the randomized controlled trial framework. All adjusted analyses included the following covariates: age, sex, years of education, APOE ε4 carrier status (positive/negative), and treatment arm. Analyses of the ADAS-Cog and CDR-SB included the corresponding baseline score as an additional covariate.

Unadjusted and adjusted partial correlations were calculated to examine relationships among baseline plasma biomarker concentrations, baseline MRI volumetric measures, and baseline scores for ADAS-Cog11 and CDR-SB using Spearman’s method. Spearman's method was used due to its robustness to non-normal distributions and outliers commonly observed in biomarker data, and to capture potentially non-linear monotone relationships between variables.

Linear regression models were used to investigate relationships between 1) baseline plasma biomarker concentrations and 48-week change on the ADAS-Cog11 and CDR-SB; 2) baseline plasma biomarker concentrations and 48-week change in the eight specified MRI volumetric measures; and 3) 48-week change in plasma biomarker concentrations and the 48-week change in MRI volumetric measures and scores on the ADAS-Cog11 and CDR-SB. Each biomarker was analyzed separately in these models. Continuous predictors and covariates were standardized before inclusion in the regression models, while outcome variables were kept in their original scales. Therefore, the reported regression coefficients allow comparison of effect sizes across various biomarkers and scales while maintaining the clinical interpretability of the outcomes. Family-wise Bonferroni *p*-value adjustment was applied for multiple comparisons and adjusted *p*-values are reported at 5% significance level.

To identify the most parsimonious subset of baseline plasma biomarkers and/or MRI volumetric measures to predict 48-week change on the ADAS-Cog11 and CDR-SB, least absolute shrinkage and selection operator (LASSO) [17] modeling for multivariable regression was used. LASSO is a statistical technique used to select the most important predictors from a larger set of variables by identifying and retaining only those variables that significantly contribute to the prediction, while eliminating less important variables. Unlike traditional regression, which includes all predictors in the model, LASSO provides more interpretable results by reducing the number of variables to those with the greatest predictive utility. Covariates were forced into the LASSO models without penalty (naïve model). Ten-fold cross validation was used for LASSO to choose the model with the minimum prediction error. R-squared was reported and compared with the defined naïve model.

#### Sensitivity analyses

Sensitivity analyses were performed to determine whether the inclusion of body mass index (BMI) [18]; kidney function [18, 19] (i.e., estimated glomerular filtration rate (eGFR)); baseline blood pressure [20, 21]; or study site altered the results of the regression models. To examine whether the relationship between baseline biomarker concentrations and 48-week outcome change differed between the treatment groups, all regression models were extended to include an interaction term between each baseline biomarker concentration and treatment arm assignment.

#### Power calculations

Informed by the results of the regression analyses, power calculations were performed to estimate the sample sizes required to detect a 30% difference between treatment and control groups in change from baseline to week 48 on the ADAS-Cog or CDR-SB with 80% or 90% power, based on various plasma biomarker concentrations. This effect size is within the range of those recently reported with lecanemab [22] and donanemab [23]. For each biomarker or combination, participants were divided into two groups based on the overall observed mean concentration of plasma biomarkers at baseline, categorized as high and low. Power calculations were replicated using the observed median to split the cohort into two groups. Two-tailed two sample t tests were used, with significance level of 0.05.

Analyses were conducted using a complete case analysis approach for all variables relevant to each specific analysis. All analyses were done using R version 4.1.3.

## Results

### Participant demographic, clinical, and biomarker characteristics

Table 1 presents baseline demographic and biomarker characteristics of the overall analysis sample (*n* = 319), the subsample with baseline and at least one clinical outcome measure at week 48 (*n* = 264), and the subsample with baseline and week-48 MRI scans of sufficient quality for analysis and registration with QUARC (*n* = 230). The flow diagram of analysis sample selection is presented in Supplemental Fig. 1. Concentrations of the five plasma biomarkers (Aβ42/40 ratio, T-tau, P-tau181, NfL, GFAP) by treatment arm at baseline and week 48 are displayed in Supplemental Fig. 2. There were no statistically significant differences between the troriluzole and placebo groups on any of the plasma biomarkers nor on MRI volumetric measures at baseline (MRI data not shown). Plots of 48-week change in concentrations of the five plasma biomarkers and eight MRI volumetric measures by treatment arm are displayed in Supplemental Fig. 3. After Bonferroni adjustment for multiple comparisons, there were no significant differences between the troriluzole and placebo groups in 48-week plasma biomarker change nor in 48-week change in MRI volumes, supporting the decision to collapse across arms.

**Table 1:** Characteristics of biomarker study participants at baseline

	Initial Plasma	Clinical 48-week Change	MRI 48-week Change
n^a^	319	264	230
*Demographics*			
Age: mean (SD)	71.52 (8.03)	71.53 (8.08)	71.47 (8.02)
Sex: N (%) male	132 (41.4)	113 (42.8)	100 (43.5)
APOE ε4 carrier: N (%) positive	213 (66.8)	181 (68.6)	163 (70.9)
Education: mean (SD)	15.28 (3.07)	15.18 (3.01)	14.98 (2.85)
Race: N (%)			
Asian	4 (1.25)	4 (1.52)	4 (1.74)
Black or African American	9 (2.82)	9 (3.41)	6 (2.61)
Native Hawaiian or Other Pacific Islander	1 (0.31)	0 (0.00)	0 (0.00)
White	305 (95.6)	251 (95.1)	220 (95.7)
Ethnicity: N (%)			
Hispanic or Latino	9 (2.82)	6 (2.27)	4 (1.74)
Not Hispanic or Latino	307 (96.2)	256 (97.0)	224 (97.4)
Not Reported	3 (0.94)	2 (0.76)	2 (0.87)
*Clinical Measurements (measured at baseline)*			
MMSE: mean (SD)	19.35 (3.84)	19.23 (3.86)	19.46 (3.83)
MoCA: mean (SD)	12.59 (4.78)	12.57 (4.68)	12.86 (4.71)
ADAS-Cog11: mean (SD)	25.97 (8.10)	26.07 (8.19)	25.69 (8.22)
CDR-SB: mean (SD)	6.46 (2.47)	6.45 (2.47)	6.40 (2.51)
*Plasma Biomarker Measurements (measured at baseline)*			
Aβ42/40: mean (SD)	0.05 (0.01)	0.05 (0.01)	0.05 (0.01)
GFAP, pg/mL: mean (SD)	399.9 (173.6)	390.0 (168.2)	390.7 (167.6)
NfL, pg/mL: mean (SD)	29.05 (11.45)	28.72 (11.38)	28.36 (10.80)
P-tau181, pg/mL: mean (SD)	4.14 (1.59)	4.04 (1.54)	4.04 (1.54)
T-tau, pg/mL: mean (SD)	2.36 (1.25)	2.34 (1.24)	2.33 (1.26)

^a^The three sample sizes reflect the number of participants from the mITT population with plasma biomarker measurements available at baseline, number of participants with 48-week change of clinical outcomes available, and number of participants with 48-week change of MRI volumetric measures available

In the combined participant sample, there was a significant increase over 48 weeks in plasma NfL (mean change = 3.10 ng/L, 95% CI [1.82, 4.37], adjusted *p*-value < 0.001) and GFAP (mean change = 45.9 ng/L, 95% CI [30.0, 61.7], adjusted *p*-value < 0.001), but no significant changes were observed in T-tau, P-tau181, or Aβ42/40. All eight MRI measures in the combined group changed significantly over the 48-week trial in the direction of increasing degeneration and volume loss (shown as mean QUARC change in cm^3^ [standard deviation]: Entorhinal: -2.25 [1.80], hippocampal: -1.19 [1.77], bilateral lateral ventricles: 11.5 [6.33], whole brain: -2.44 [1.93], precuneus: -2.66 [1.71], isthmus: -2.39 [1.51], mid-temporal: -3.50 [1.94], and supramarginal: -2.77 [2.13]; all adjusted *p*-values < 0.001). ADAS-Cog and CDR-SB scores worsened significantly over the 48-week trial (6.21 [7.80] and 1.97 [2.39] point increase, respectively; *p*-values< 0.001).

### Association of baseline plasma biomarker concentrations with baseline clinical outcome and MRI volumetric measures

**Fig. 1:**
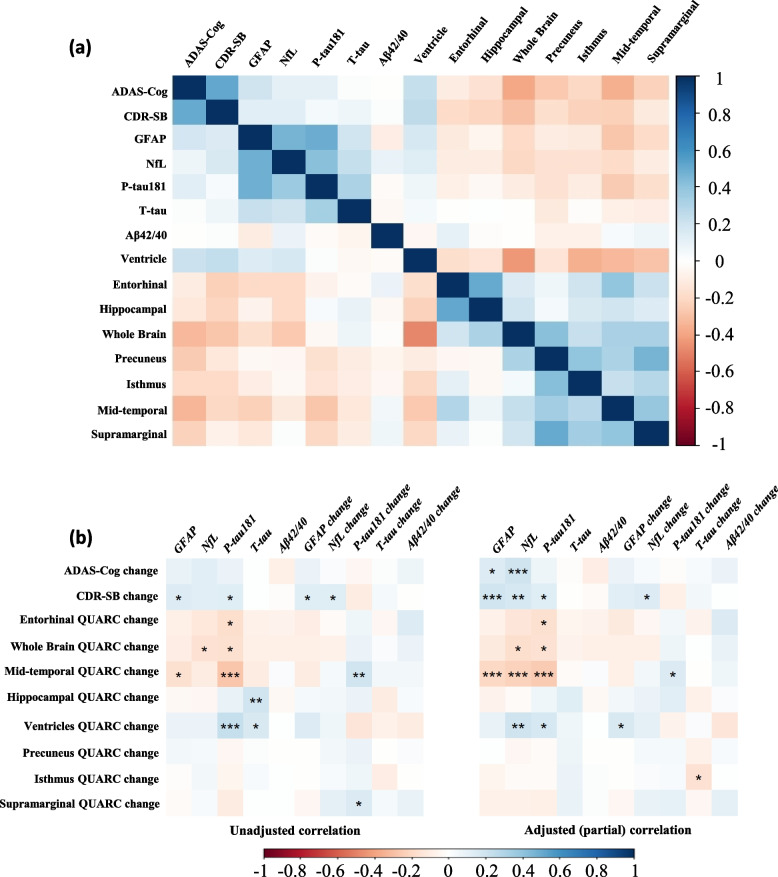
Heat maps of (**a**) Unadjusted (lower triangle) and adjusted (partial; upper triangle) correlations between baseline plasma biomarker concentrations, baseline MRI volumetric measures, and baseline scores on the ADAS-Cog11 and CDR-SB, and (**b**) Unadjusted and adjusted (partial) correlations between baseline or 48-week change of plasma biomarker concentrations, with 48-week change on the ADAS-Cog11, CDR-SB, and MRI volumetric measures. * *p* < 0.05; ** *p* < 0.01; *** *p* < 0.005

Adjusted partial correlation coefficients describing the association between baseline plasma biomarker concentrations and baseline MRI volumetric and ADAS-Cog11 and CDR-SB measures are shown in Fig. 1a. Partial correlations (upper triangle in Fig. 1a) were highly consistent with unadjusted correlations (lower triangle in Fig. 1a), indicating that relationships among these measures were not substantially influenced by age, sex, years of education, APOE ε4 carrier status, or treatment assignment. Baseline plasma GFAP, NfL, and P-tau181 concentrations were positively associated with baseline ADAS-Cog11, CDR-SB, and eight MRI ventricular volume ($\rho$ from 0.02 to 0.18, p from 0.001 to 0.770), and negatively associated with baseline whole brain and regional cortical volumes ($\rho$ from -0.04 to -0.23, p from < 0.001 to 0.580). Detailed correlations can be found in Supplemental Table 2A.

### Association of baseline plasma biomarker concentrations with longitudinal change in clinical outcome measures

Figure 1b presents unadjusted and adjusted (partial) correlation coefficients describing the association between baseline concentration of each plasma biomarker and 48-week change in ADAS-Cog11 and CDR-SB scores. Correlation analysis indicated that higher baseline plasma NfL and GFAP were associated with worsening over 48-week on the ADAS-Cog11 ($\rho$ = 0.20 with 95% CI [0.07, 0.33] and 0.14 with 95% CI [0.01, 0.27], *p* = 0.002 and 0.027, respectively) and CDR-SB ($\rho$ = 0.18 with 95% CI [0.05, 0.29] and 0.20 with 95% CI [0.07, 0.31], *p* = 0.006 and 0.002, respectively) after adjusting for relevant covariates. Similarly, higher baseline plasma P-tau181 was correlated with worsening over 48-weeks on the CDR-SB ($\rho$ = 0.13 with 95% CI [0.01, 0.26], *p* = 0.043). Baseline T-tau and Aβ42/40 ratio were not significantly associated with 48-week change in the clinical outcome measures. Detailed correlations can be found in Supplemental Table 2B.

**Fig. 2:**
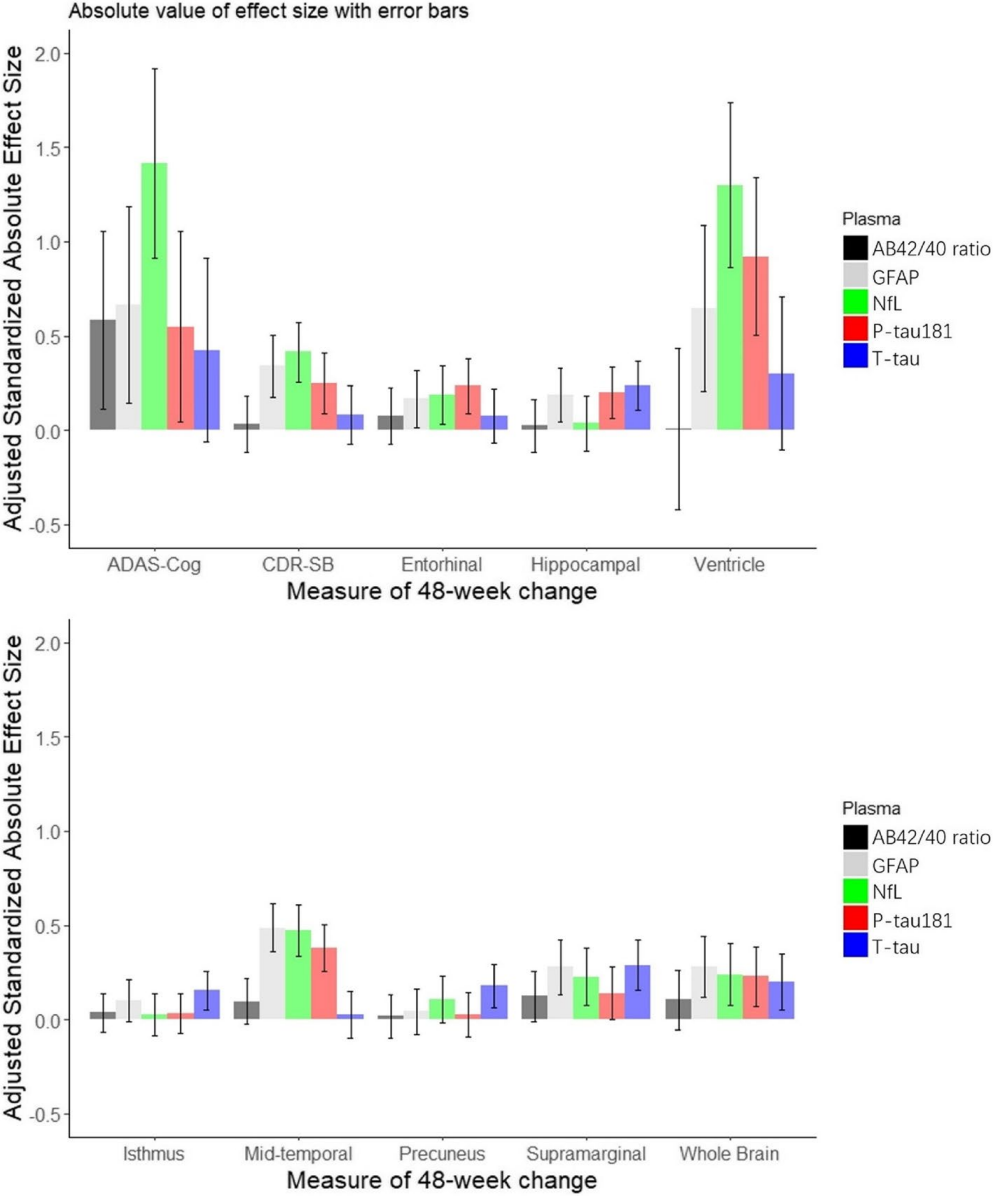
Adjusted standardized effect sizes (as absolute values) for the association of each plasma biomarker at baseline with 48-week change on ADAS-Cog11, CDR-SB, and MRI volumetric measures. Error bars indicate the standard error of the estimated effect size

Results of linear regression models testing whether the concentration of each plasma biomarker at baseline predicts 48-week change in ADAS-Cog11 or CDR-SB score are presented in Table 2. Only baseline plasma NfL remained a significant predictor of worsening on the ADAS-Cog11 and CDR-SB after adjusting for covariates. A one standard deviation higher baseline plasma NfL concentration was associated with a 1.42 point (95% CI [0.43, 2.41]; *p* = 0.005) increase (i.e., worsening) on the ADAS-Cog11 or a 0.42-point (95% CI [0.10, 0.73], *p* = 0.010) increase (i.e., worsening) in CDR-SB. Adjusted standardized effect sizes (as absolute values) for each baseline plasma biomarker’s association with 48-week change in ADAS-Cog11 and CDR-SB scores are shown in Fig. 2.

**Table 2:** Linear regression models predicting 48-week change on the ADAS-Cog11 (2A) or CDR-SB (2B) by baseline value of each plasma biomarker after adjusting for age, sex, years of education, APOE ε4, treatment arm, and baseline score

(2A) Predicting ADAS-Cog change							
Plasma biomarker	Std. coefficient	SE	t value	*P* value	95% CI Lower.bound	95% CI Upper.bound	Bonferroni adjusted *P* value
GFAP	0.66	0.52	1.28	0.204	−0.36	1.69	1.000
NfL	1.42	0.50	2.82	0.005	0.43	2.41	0.026
P-tau181	0.55	0.51	1.08	0.280	−0.45	1.54	1.000
T-tau	−0.43	0.49	−0.87	0.383	−1.38	0.53	1.000
Aβ42/40	−0.59	0.47	−1.24	0.216	−1.51	0.34	1.000
(2B) Predicting CDR-SB change							
Plasma biomarker	Std. coefficient	SE	t value	*P* value	95% CI Lower.bound	95% CI Upper.bound	Bonferroni adjusted *P* value
GFAP	0.34	0.16	2.09	0.038	0.02	0.66	0.190
NfL	0.42	0.16	2.61	0.010	0.10	0.73	0.048
P-tau181	0.25	0.16	1.57	0.118	−0.06	0.56	0.589
T-tau	−0.08	0.16	−0.52	0.605	−0.39	0.23	1.000
Aβ42/40	−0.03	0.15	−0.22	0.829	−0.33	0.26	1.000

The increase in adjusted R-squared associated with different combinations of plasma biomarkers in regression models is shown in Supplemental Table 4. Multivariable LASSO regression revealed that a combination of baseline plasma NfL, T-tau, and the Aβ42/40 ratio best predicted worsening over 48-weeks on the ADAS-Cog11. The adjusted R-square of the model increased by 7% (from 4.3% to 7.4%) when NfL was added to a model that contained age, sex, years of education, APOE ε4, treatment arm, baseline ADAS-Cog11 score, baseline T-tau and baseline Aβ42/40 ratio (Supplemental Table 4 A). Baseline NfL alone best predicted worsening over 48-weeks on CDR-SB; no other baseline plasma biomarkers were selected. Adjusted R-square increased by 50% (from 3.6% to 5.4) when NfL was added to the adjusted model (Supplemental Table 4B).

### Association of baseline plasma biomarker concentrations with longitudinal change in MRI volumetric measures

Figure 1b also presents unadjusted and adjusted (partial) correlation coefficients describing the association between the baseline concentration of each plasma biomarker and 48-week change in MRI volumetric measures determined by the QUARC non-linear serial MRI registration method. Higher concentrations of baseline plasma NfL and P-tau181 were associated with greater loss of whole brain volume ($\rho$ = -0.18 with 95% CI [-0.32, -0.04], *p* = 0.019 and $\rho$ = -0.16 with 95% CI [-0.33, -0.01], *p* = 0.036, respectively) and greater increase in volume of the lateral ventricles ($\rho$ = 0.21 with 95% CI [0.06, 0.34], *p* = 0.006 and $\rho$ = 0.17 with 95% CI [0.02, 0.30], *p* = 0.024, respectively) over the 48-week trial. Higher baseline P-tau181 was also associated with greater decrease in entorhinal volume ($\rho$ = -0.16 with 95% CI [-0.31, -0.01], *p* = 0.031); and higher concentrations of baseline plasma GFAP, NfL, and P-tau181 were associated with greater decrease in middle-temporal gyrus volume ($\rho$ = -0.21 with 95% CI [-0.34, -0.07], *p* = 0.002; $\rho$ = -0.23 with 95% CI [-0.36, -0.08], *p* = 0.001; $\rho$ = -0.25 with 95% CI [-0.38, -0.13], *p* < 0.001, respectively). Baseline plasma biomarkers were not significantly associated with change in the other cortical ROIs (see Fig. 1b). Baseline T-tau and Aβ42/40 ratio were not significantly associated with change in any brain volumetric measure. Detailed correlation matrix can be found in Supplemental Table 2B.

Results from linear regression models testing whether baseline concentration of each plasma biomarker would predict 48-week MRI volumetric change are presented in Table 3 and Supplemental Table 3. Baseline plasma NfL concentration was a significant predictor of 48-week increase in lateral ventricular volume (mean change 1.30 cm^3^ with 95% CI [0.43, 2.17], *p* = 0.004), and baseline concentration of plasma NfL, P-tau181, and GFAP significantly predicted 48-week decrease in middle-temporal gyrus volume (mean change in cm^3^ with 95% CI: -0.47 [-0.74, -0.20], -0.38 [-0.63, -0.13], -0.49 [-0.74, -0.23], all *p* < 0.010; Table 3). Results of regression models for the remaining ROIs, which were not significantly predicted by baseline biomarker concentration, are provided in Supplemental Table 3. Adjusted standardized effect sizes (as absolute values) for each baseline plasma biomarker’s prediction of 48-week change in each MRI volumetric measure are shown in Fig. 2.

**Table 3:** Linear regression models predicting 48-week QUARC change on bilateral lateral ventricles and mid-temporal volumes by baseline value of each plasma biomarker after adjusting for age, sex, years of education, APOE ε4 status, treatment arm, and baseline ADAS-Cog11 score

	Std. coefficient	SE	t value	*P* value	95% CI Lower.bound	95% CI Upper.bound	Bonferroni adjusted *P* value
*Bilateral Lateral Ventricles volume 48-week QUARC change*							
GFAP	0.64	0.44	1.46	0.146	−0.23	1.51	0.728
NfL^**^	1.30	0.44	2.96	0.004	0.43	2.17	0.018
P-tau181^*^	0.92	0.42	2.20	0.030	0.09	1.75	0.148
T-tau	0.30	0.41	0.74	0.463	−0.50	1.10	1.000
Aβ42/40	0.01	0.43	0.02	0.983	−0.84	0.85	1.000
*Mid-temporal volume 48-week QUARC change*							
GFAP^**^	−0.49	0.13	−3.78	0.000	−0.74	−0.23	0.001
NfL^**^	−0.47	0.14	−3.47	0.001	−0.74	−0.20	0.003
P-tau181^**^	−0.38	0.13	−3.05	0.003	−0.63	−0.13	0.013
T-tau	0.03	0.12	0.21	0.835	−0.22	0.27	1.000
Aβ42/40	0.10	0.12	0.79	0.432	−0.15	0.34	1.000

^*^Indicates the unadjusted *p*-value was significant (*p* < 0.05)

^**^indicates the family-wise Bonferroni adjusted *p*-value was significant (*p* < 0.05)

### Prediction of clinical outcomes from baseline plasma biomarker and MRI volumetric measures

Multivariable LASSO regression was performed to select parsimonious subsets of baseline plasma biomarker and MRI brain measurements that best predict clinical change over 48 weeks. Separate analyses were performed for 48-week change in ADAS-Cog11 and CDR-SB scores. The full prediction models are presented in Supplemental Table 5. Baseline plasma NfL concentration, whole brain volume, and precuneus cortex volume were selected by LASSO for predicting 48-week worsening on the ADAS-Cog11. The adjusted R-squared of the model increased substantially, from 5.1% to 13.5%, when these three baseline biomarkers were added to the naïve model (Supplemental Table 5A). For predicting 48-week change on the CDR-SB, baseline plasma NfL concentration and six MRI measurements (whole brain, lateral ventricles, precuneus cortex, isthmus of cingulate gyrus, middle-temporal gyrus, supramarginal gyrus) were selected by LASSO, increasing the adjusted R-squared of the naïve model from 3.6% to 10.8% (Supplemental Table 5B).

### Association of 48-week change in plasma biomarkers with longitudinal change in clinical outcome measures

There was no significant association between 48-week change on any plasma biomarker and worsening on the ADAS-Cog11 (Supplemental Table 6A). Increase in plasma NfL concentration (but no other plasma biomarker) was significantly associated with worsening on the CDR-SB score after adjusting for covariates and multiple comparisons (Standardized Coefficient = 0.43, 95% CI = [0.12, 0.74], *p* = 0.007, Bonferroni adjusted *p* = 0.033, Supplemental Table 6B). That is, a 1 standard deviation increase in the rate of NfL change over 48 weeks was associated with a 0.43 increase in CDR-SB score.

### Sensitivity analyses

The inclusion of BMI, eGFR, blood pressure, or study site did not significantly alter the results of the regression analyses. Similarly, in separate models including an interaction term between each baseline biomarker concentration and treatment arm assignment, none of the interaction terms was statistically significant, and the model results were not changed.

### Utility of plasma biomarkers for informing sample size calculations in prospective RCTs

Based on the finding that baseline plasma NfL was consistently and highly predictive of clinical worsening over the 48-week trial, we opted to explore the potential benefit of considering baseline NfL, alone and in combination with Aβ42/40 and P-tau181, when determining requisite sample size for statistical power in a randomized controlled trial. Participants were divided into two groups based on the overall observed mean NfL concentration at baseline (29.1 ng/L). The high and low NfL groups did not differ significantly on the MMSE, ADAS-Cog, or CDR-SB at baseline (all *p*-values > 0.10). Nevertheless, over 48 weeks, the group with relatively high baseline NfL exhibited significantly more rapid worsening on the ADAS-Cog than the group with relatively low baseline NfL (mean ADAS-Cog change +7.14 points (SD = 6.92) and +5.79 points (SD = 7.95), respectively). Similar results were observed for change in CDR-SB over 48 weeks. Dichotomizing the groups at the observed median NfL concentration at baseline (27.2 ng/L), rather than the mean, did not significantly alter the results.

Table 4 shows requisite sample sizes to detect a 30% difference between treatment and control groups in change from baseline to week 48 on the ADAS-Cog and the CDR-SB with 80% power, given entry criteria based on baseline concentrations of plasma biomarkers including NFL. Results show that a trial including only participants with relatively high baseline plasma NfL (above 29.1 ng/L in our sample) would require approximately 40% fewer participants than a trial that did not consider baseline NfL for inclusion (*N* = 330 vs. 552; Table 4), assuming treatment efficacy is equal for high- and low-NfL patients. When relatively high NfL is added to low Aβ42/40 and high P-tau181 concentrations as an inclusion criterion, the requisite sample size is reduced by half compared to when using only low Aβ42/40 and high P-tau181 (*N* = 276 vs. 552). Additional analyses examining NfL stratification specifically within participants showing AD-consistent biomarker profiles (low Aβ42/40 + high P-tau181) suggested similar potential for sample size reduction (*N* = 276 vs. 608). These results suggest that baseline plasma NfL may be useful for enriching recruitment into AD clinical trials, above and beyond plasma Aβ42/40 and P-tau181.

**Table 4:** Plasma biomarker-informed sample size calculations. Tabled values reflect sample size required to detect a 30% difference between treatment and control groups in change from baseline to week 48 on the ADAS-Cog or CDR-SB. Biomarker high and low groups were defined by dividing the cohort at the observed baseline mean value. The total sample size required for an adequately powered 2-group randomized controlled trial is reported

	Outcome Measure			
	ADAS-Cog		CDR-SB	
Inclusion criteria for trial entry	Required *n* for 80% power	Required *n* for 90% power	Required *n* for 80% power	Required *n* for 90% power
No biomarker inclusion criteria	552	740	518	692
NfL high (> 29.07 ng/L)	330	442	348	464
NfL low (< = 29.07 ng/L)	630	884	694	928
Aβ42/40 low (< 0.053)	426	568	500	668
Aβ42/40 high (> = 0.053)	556	742	538	718
p-tau181 high (> 4.14 ng/L)	456	610	436	582
p-tau181 low (< = 4.14 ng/L)	512	686	584	782
Aβ42/40 low + p-tau181 high	404	542	398	532
Aβ42/40 high or p-tau181 low	510	682	550	736
Aβ42/40 low + p-tau181 high + NfL high	276	368	282	376
Aβ42/40 low + p-tau181 high + NfL low	608	812	690	922

## Discussion

To explore the potential of plasma biomarkers to inform and enhance design of future clinical trials for mild-to-moderate dementia due to AD, we retrospectively evaluated the relationship between plasma biomarkers and clinical and neuroimaging outcome measures in a 48-week, phase 2 RCT of troriluzole. Results revealed that baseline plasma NfL was a significant predictor of more rapid worsening on the ADAS-Cog11 and CDR-SB over the 48-week trial, whereas baseline concentrations of P-tau181, T-tau, Abeta42/40 ratio, or GFAP were not significantly associated with change on these outcomes (Table 2). Further, longitudinal increase in plasma NfL over the course of the 48-week trial was significantly associated with worsening on the CDR-SB, whereas 48-week change in plasma Aβ42/40, T-tau, P-tau181, or GFAP was not associated with clinical change. Higher baseline NfL predicted increasing ventricular and decreasing mid-temporal cortical volumes, but was not significantly predictive of changes in hippocampus, entorhinal cortex, precuneus cortex, isthmus of cingulate gyrus, supramarginal gyrus, or whole brain volumes. Sensitivity analyses confirmed that inclusion of eGFR, BMI, blood pressure, or study site did not significantly alter the observed associations between plasma biomarkers and clinical or MRI outcomes in this cohort. Results suggest that plasma NfL, a marker of axonal degeneration that is not specific to AD [24, 25], may nevertheless enhance clinical trial design in mild-to-moderate dementia due to AD, perhaps by identifying participants who are at risk of more rapid worsening.

We used LASSO multivariable regression as a statistical approach because it selects the most parsimonious set of predictors and provides greater prediction accuracy than other regression models, therein enhancing model interpretation. From the full set of baseline plasma biomarkers (i.e., Aβ42/40, T-tau, P-tau181, NfL, and GFAP), LASSO regression selected baseline plasma NfL for inclusion in all models predicting clinical change. Specifically, plasma NfL was the only significant predictor of 48-week change on the CDR-SB and the most significant among the selected combination of plasma biomarkers (NfL, T-tau, and Aβ42/40 ratio) to predict change in ADAS-Cog. Similarly, among analyses that included baseline MRI volumes in addition to plasma biomarkers as potential predictors, plasma NfL was the only plasma biomarker retained in the final LASSO models. These results indicate that plasma NfL has significant utility as an independent predictor of clinical worsening in patients with mild-to-moderate AD.

Results of our correlation analyses revealed significant cross-sectional relationships between baseline plasma NfL and measures of disease severity. While participants above versus below the mean NfL value did not differ significantly on baseline MMSE, ADAS-Cog11, or CDR-SB when dichotomized into two groups for our power calculations, this may be due to the loss of statistical power when analyzing NfL as a dichotomous rather than continuous variable. The significant cross-sectional associations in our correlation analyses are consistent with the established literature demonstrating the relationship of NfL with disease severity in neurodegenerative conditions [25], further validating the biomarker's relevance in our cohort.

We used power calculations to further explore the potential utility of NfL, alone and in combination with Aβ42/40 and P-tau181. Results of this exploratory modeling suggest that considering NfL at inclusion could have reduced the requisite sample size by up to 40%. These power estimates demonstrate the potential utility of considering baseline NfL in trial design; however, some limitations are worth noting. First, because the high/low biomarker groups were created by splitting the T2-Protect-AD cohort at the observed mean, they are potentially cohort specific. Second, when comparing high vs. low NfL among those with low Aβ42/40 and high p-tau181, the sample size becomes relatively small, which may affect the reliability of the power calculations. Therefore, while these results are promising, they should be validated in larger cohorts. More generally, while considering NfL concentration at inclusion may enhance statistical power, doing so may limit generalizability of trial results. Further, to the extent that higher concentration of NfL is associated with a more advanced stage of neurodegeneration, selecting participants with relatively high NfL may make demonstrating a clinical treatment effect more difficult. Increasingly AD trials are focused on early-stage disease, when disease modifying therapies are more likely to demonstrate therapeutic efficacy. Our results may not, however, apply to MCI or preclinical AD.

Our results are consistent with previous work supporting the potential utility of plasma NfL as a sensitive and dynamic biomarker in MCI and dementia due to AD. In ADNI, for example, plasma NfL was increased at baseline in participants diagnosed with MCI and AD dementia relative to cognitively unimpaired older adults, and it showed the greatest increase over time in participants with dementia due to AD [10]. Longitudinally, faster increase in plasma NfL was associated with accelerated reduction in FDG-PET measures, increase of ventricular volume, and worsening scores on the MMSE, CDR, and ADAS-Cog [10]. Secondary analyses of RCT data have similarly linked changes in NfL levels with changes in cognitive-functional composite outcomes [26, 27] and whole brain volume loss [27].

Other retrospective analyses of RCT data have also demonstrated the prognostic utility of NfL. For example, in a small sub-sample of older adults with mild-to-moderate AD dementia participating in the FIT-AD Trial, higher baseline plasma NfL was associated with greater rate of decline of cognition and function over the 12-month trial, and greater increase in plasma NfL was associated with greater cognitive and functional decline [9]. Similarly, post-hoc analyses of biomarker data from a phase 3 trial in mild probable AD (MMSE 20–26), revealed that baseline plasma NfL was significantly associated with change over the course of the18-month trial on clinical scales of cognition and function, whereas no such association was observed for T-tau [28]. These authors reported that the inclusion of baseline NfL did not significantly increase estimated power to detect change in ADAS-Cog total scores over the 18-month trial; however, results from their power estimates may have differed from ours due to differences in cohort, trial duration, or model parameters. In contrast, Mendes and colleagues [29] estimated that adding NfL to the inclusion criteria for an AD trial in amyloid-PET positive MCI participants could reduce the sample size by up to 25% and in participants with amyloid-PET and tau-PET positivity sample size could be reduced by as much as 50%. Our results similarly support the potential utility of NfL and extend this finding to trials of mild-to-moderate AD dementia.

In patients with AD, increased concentrations of NfL in plasma or serum are associated with volume loss on structural MRI, particularly in the brain regions typically affected by AD; glucose hypometabolism on FDG-PET; and loss of white matter integrity on diffusion tensor imaging [30]. NfL is, however, a nonspecific marker of neuroaxonal integrity [24, 25], and it is unclear how NfL relates to amyloid or tau pathology in AD. The biological mechanisms mediating the observed associations between rates of clinical decline with higher concentrations of NfL in AD have yet to be fully elucidated. This is an important direction for future investigation, which may lead to novel therapeutic interventions. Increasing neurofilament light has been associated with imaging and clinical features of cerebral small vessel disease (SVD) [21] and risk of cognitive decline associated with SVD [20]. Comorbid SVD is unlikely to have been a major contributor to the observed results in our study, however, since by protocol patients with clinical of imaging evidence of significant SVD or other significant cerebrovascular disease were excluded from participation in the T2 Protect AD trial (see Study Design and Participants).

### Limitations

Several limitations of this study should be noted. First, participants in the T2 Protect AD trial were enrolled based on a clinical diagnosis of mild-to-moderate probable AD by NIA-AA criteria [13]; no biomarker confirmation of AD pathology was required. Nevertheless, in a subsample of participants who consented to a voluntary research lumbar puncture, the prevalence of biomarker positivity was 94%, suggesting a high degree of diagnostic accuracy among the trial participants. Second, measures of tau that have been shown to be sensitive and dynamic markers of AD diagnosis and disease progression, such as plasma P-tau217, tau PET, and MBTR243 tau, were not available for comparison. Third, T2 Protect AD participants were predominantly White, non-Hispanic, and had, on average, some college education; therefore, caution is warranted in generalizing these biomarker findings to more racially, ethnically, or socially diverse cohorts [31]. Fourth, because our findings were derived using data from a trial of mild-to-moderate AD, they may not apply to earlier stages of the disease. Finally, we were not able to examine change in plasma biomarkers as a function of treatment response in this negative trial.

## Conclusions

Post-hoc analyses of data from a phase 2 RCT in mild-to-moderate AD demonstrated that baseline plasma NfL predicted worsening clinical outcomes and MRI volumes over the 48-week trial better than baseline Aβ42/40, T-tau, P-tau181, or GFAP. Further, increasing plasma NfL concentration over the course of the trial was associated with increasing clinical impairment. Plasma NfL is an easily accessible biomarker that may enhance trial design in mild-to-moderate dementia due to AD.

## Supplementary Information

Supplementary Material 1.

## Data Availability

Deidentified participant data are available to qualified researchers for academic and non-commercial use through a signed data access agreement.
