# Supplementary material for: Prognostic value of plasma biomarkers for informing clinical trial design in mild-to-moderate Alzheimer’s disease
Source: Alzheimers Res Ther. 2025 May 2;17:97. doi: 10.1186/s13195-025-01745-3 (PMC12046789; doi:10.1186/s13195-025-01745-3)

**Supplemental Material**

**Supplemental Table 1** Quality assurance and quality control specifications for each analyte.

**Supplemental Table 2 (A)** Correlations of unadjusted (lower triangle) and adjusted (partial; upper triangle) correlations between baseline plasma biomarker concentrations, baseline MRI volumetric measures, and baseline scores on the ADAS-Cog11 and CDR-SB. **(B)** Unadjusted (B1) and adjusted/partial (B2) correlations between baseline or 48-week change of plasma biomarker concentrations, with 48-week change on the ADAS-Cog11, CDR-SB, and MRI volumetric measures.

**Supplemental Table 3** Results of linear regression models predicting 48-week QUARC change for ROIs not significantly predicted by baseline plasma biomarker concentrations after adjusting for age, sex, years of education, APOE ε4 status, treatment arm, and baseline ADAS-Cog11 score.

**Supplemental Table 4** Comparing adjusted R-squared among LASSO selected model, combinations of LASSO selected biomarkers, and univariate biomarker models.

**Supplemental Table 5** Multivariable LASSO regression selected models predicting 48-week change in ADAS-Cog11 **(5A)** or CDR-SB score **(5B)**. Potential predictors included baseline plasma biomarkers and MRI volumes. Age, sex, education, APOE, arm assignment, and baseline score were forced into the LASSO models without penalty (naïve model).

**Supplemental Table 6** Linear regression models testing whether 48-week change in each plasma biomarker was associated with 48-week change in ADAS-Cog11 **(6A)** or CDR-SB **(6B)** score.

**Supplemental Figure 1** Flow diagram reflecting the number of available participants or samples for analyses.

**Supplemental Figure 2** Boxplots with spaghetti lines showing baseline and 48-week concentrations of GFAP, NfL, p-tau181, T-tau, and Aβ42/40 ratio as a function of treatment arm (i.e., troriluzole vs. placebo).

**Supplemental Figure 3** Raincloud plots showing distributions of **(a)** 48-week change for five plasma biomarkers (Aβ42/40 ratio, T-tau, p-tau181, NfL, GFAP) and **(b)** 48-week QUARC change for eight MRI volumetric measures (hippocampus, whole brain, lateral ventricles, entorhinal cortex, precuneus cortex, isthmus of cingulate gyrus, middle-temporal gyrus, supramarginal gyrus) as a function of treatment arm (i.e., troriluzole vs. placebo).

**Supplemental Table 1** Quality assurance and quality control specifications for each analyte

| Analyte | Calibrators | | | | Kit Controls | | | | Matrix Control | | Plasma |
| --- | --- | --- | --- | --- | --- | --- | --- | --- | --- | --- | --- |
|  | % error | % recovery | Intra-well CV | Inter-run CV | % error | % recovery | Intra-well CV | Inter-run CV | Intra-well CV | Inter-run CV | Intra-well CV |
| NfL | 5 | 100 | 5 | 6.2 | 9.6 | 101 | 6 | 13 | 4.4 | 7.9 | 3.9 |
| GFAP | 2.7 | 101 | 6.0 | 3.1 | 11.8 | 99.5 | 6 | 10 | 6.9 | 8.3 | 7.4 |
| P-Tau 181 | 6.0 | 102 | 5.0 | 4.6 | 9.2 | 91 | 4.9 | 6.7 | 6.6 | 11.6 | 5.3 |
| T-tau | 3.5 | 100 | 5.7 | 4.4 | 12 | 91 | 5.7 | 8.8 | 5.6 | 16 | 7.2 |
| Aβ40 | 5.8 | 102 | 4.2 | 7.5 | 6.5 | 96 | 4.8 | 6.3 | 5.4 | 9.6 | 4.0 |
| Aβ42 | 4.7 | 99 | 4.0 | 5.0 | 9.0 | 95 | 4.6 | 11 | 3.2 | 11 | 4.1 |

**Supplemental Table 2 (A)** Correlations of unadjusted (lower triangle) and adjusted (partial; upper triangle) correlations between baseline plasma biomarker concentrations, baseline MRI volumetric measures, and baseline scores on the ADAS-Cog11 and CDR-SB. **(B)** Unadjusted (B1) and adjusted/partial (B2) correlations between baseline or 48-week change of plasma biomarker concentrations with 48-week change on the ADAS-Cog11, CDR-SB, and MRI volumetric measures.

| (A) | ADAS-Cog score | CDR-SB score | Plasma GFAP (pg/mL) | Plasma NfL (pg/mL) | Plasma P-tau181 (pg/mL) | Plasma T-tau (pg/mL) | Plasma Abeta42/40 ratio | Bilateral Lateral Ventricles volume | Entorhinal volume | Hippocampal volume | Whole Brain volume | Precuneus volume | Isthmus volume | Mid-temporal volume | Supramarginal volume |
| --- | --- | --- | --- | --- | --- | --- | --- | --- | --- | --- | --- | --- | --- | --- | --- |
| ADAS-Cog score | 1 | 0.519*** | 0.209*** | 0.114* | 0.116* | 0.01 | -0.009 | 0.240*** | -0.11 | -0.158* | -0.384*** | -0.267*** | -0.201** | -0.352*** | -0.225*** |
| CDR-SB score | 0.502*** | 1 | 0.128* | 0.124* | 0.045 | 0.06 | 0.019 | 0.259*** | -0.184** | -0.215*** | -0.295*** | -0.176** | -0.221*** | -0.239*** | -0.117 |
| Plasma GFAP (pg/mL) | 0.183** | 0.156** | 1 | 0.469*** | 0.498*** | 0.196*** | -0.095 | 0.178** | -0.111 | -0.05 | -0.195** | -0.106 | -0.113 | -0.270*** | -0.189** |
| Plasma NfL (pg/mL) | 0.074 | 0.164** | 0.485*** | 1 | 0.420*** | 0.239*** | 0.094 | 0.138* | -0.107 | -0.109 | -0.204** | -0.156* | -0.159* | -0.183** | -0.103 |
| Plasma P-tau181 (pg/mL) | 0.122* | 0.04 | 0.485*** | 0.363*** | 1 | 0.318*** | -0.022 | 0.065 | -0.082 | -0.033 | -0.104 | -0.159* | -0.102 | -0.256*** | -0.179** |
| Plasma T-tau (pg/mL) | 0.017 | 0.06 | 0.221*** | 0.205*** | 0.330*** | 1 | -0.03 | 0.044 | -0.003 | 0.007 | -0.002 | -0.114 | -0.013 | -0.085 | -0.093 |
| Plasma Abeta42/40 ratio | -0.006 | 0.018 | -0.103 | 0.084 | -0.027 | -0.05 | 1 | -0.04 | 0.103 | -0.01 | -0.008 | -0.077 | -0.073 | 0.037 | 0.065 |
| Bilateral Lateral Ventricles volume | 0.218*** | 0.240*** | 0.149* | 0.171** | 0.02 | -0.034 | -0.027 | 1 | -0.172** | -0.144* | -0.434*** | -0.150* | -0.352*** | -0.327*** | -0.283*** |
| Entorhinal volume | -0.104 | -0.235*** | -0.196** | -0.194** | -0.066 | -0.021 | 0.085 | -0.170** | 1 | 0.507*** | 0.159* | 0.063 | 0.203** | 0.395*** | 0.215*** |
| Hippocampal volume | -0.129* | -0.218*** | -0.067 | -0.196** | 0.035 | 0.098 | -0.035 | -0.223*** | 0.512*** | 1 | 0.199** | 0.049 | 0.160* | 0.190** | 0.142* |
| Whole Brain volume | -0.329*** | -0.278*** | -0.173** | -0.265*** | -0.037 | 0.078 | -0.013 | -0.482*** | 0.196** | 0.316*** | 1 | 0.416*** | 0.228*** | 0.323*** | 0.328*** |
| Precuneus volume | -0.253*** | -0.127 | -0.039 | -0.043 | -0.169* | -0.11 | -0.05 | -0.107 | -0.04 | -0.038 | 0.310*** | 1 | 0.399*** | 0.320*** | 0.463*** |
| Isthmus volume | -0.197** | -0.193** | -0.092 | -0.039 | -0.148* | -0.1 | -0.046 | -0.208** | 0.111 | -0.031 | 0.047 | 0.424*** | 1 | 0.228*** | 0.285*** |
| Mid-temporal volume | -0.334*** | -0.208** | -0.233*** | -0.119 | -0.274*** | -0.124 | 0.059 | -0.269*** | 0.298*** | 0.073 | 0.239*** | 0.342*** | 0.273*** | 1 | 0.388*** |
| Supramarginal volume | -0.225*** | -0.078 | -0.123 | 0.014 | -0.201** | -0.103 | 0.069 | -0.207** | 0.098 | 0.019 | 0.194** | 0.502*** | 0.347*** | 0.398*** | 1 |

This table corresponds to the heatmap in Figure 1a. * indicates p<0.05, ** indicates p<0.01, *** indicates p<0.001.

| (B1) Correlation | Baseline  GFAP | Baseline  NfL | Baseline  P-tau181 | Baseline  T-tau | Baseline  Abeta42/40 | GFAP  change | NfL change | P-tau181 change | T-tau change | Abeta42/40 change |
| --- | --- | --- | --- | --- | --- | --- | --- | --- | --- | --- |
| ADAS-Cog change | 0.094 | 0.122 | 0.082 | 0.006 | -0.087 | 0.087 | 0.028 | -0.048 | 0.016 | 0.080 |
| CDR-SB change | 0.148* | 0.123 | 0.133* | 0.008 | -0.016 | 0.133* | 0.140* | -0.108 | 0.044 | -0.002 |
| Entorhinal volume QUARC change | -0.087 | -0.125 | -0.173* | -0.074 | -0.063 | -0.095 | -0.029 | 0.069 | -0.041 | 0.145 |
| Whole Brain volume QUARC change | -0.100 | -0.152* | -0.162* | -0.084 | -0.080 | -0.082 | -0.078 | 0.088 | 0.028 | 0.083 |
| Mid-temporal volume QUARC change | -0.169* | -0.106 | -0.277*** | -0.109 | 0.024 | -0.100 | 0.050 | 0.192** | 0.055 | 0.052 |
| Hippocampal volume QUARC change | -0.035 | -0.041 | 0.097 | 0.195** | -0.036 | 0.041 | 0.058 | 0.083 | -0.101 | 0.031 |
| Bilateral Lateral Ventricles volume QUARC change | 0.087 | 0.081 | 0.230*** | 0.169* | 0.010 | 0.143 | 0.068 | -0.136 | -0.078 | -0.100 |
| Precuneus volume QUARC change | 0.052 | 0.049 | -0.044 | 0.014 | -0.009 | -0.002 | 0.059 | 0.088 | -0.002 | 0.028 |
| Isthmus volume QUARC change | -0.013 | 0.055 | -0.051 | 0.037 | 0.001 | -0.006 | 0.033 | 0.067 | -0.097 | -0.003 |
| Supramarginal volume QUARC change | -0.023 | 0.037 | -0.105 | 0.005 | 0.007 | -0.042 | 0.111 | 0.158* | 0.041 | 0.096 |

This table corresponds to the left heatmap in Figure 1b. * indicates p<0.05, ** indicates p<0.01, *** indicates p<0.001.

| (B2) Partial correlation | Baseline  GFAP | Baseline  NfL | Baseline  P-tau181 | Baseline  T-tau | Baseline  Abeta42/40 | GFAP  change | NfL change | P-tau181 change | T-tau change | Abeta42/40 change |
| --- | --- | --- | --- | --- | --- | --- | --- | --- | --- | --- |
| ADAS-Cog change | 0.144* | 0.202*** | 0.074 | -0.016 | -0.095 | 0.089 | 0.038 | -0.011 | 0.037 | 0.088 |
| CDR-SB change | 0.196*** | 0.176** | 0.131* | -0.005 | -0.022 | 0.125 | 0.143* | -0.087 | 0.056 | 0.007 |
| Entorhinal volume QUARC change | -0.078 | -0.135 | -0.163* | -0.053 | -0.063 | -0.111 | -0.041 | 0.052 | -0.064 | 0.151 |
| Whole Brain volume QUARC change | -0.114 | -0.177* | -0.159* | -0.069 | -0.082 | -0.086 | -0.082 | 0.072 | 0.002 | 0.091 |
| Mid-temporal volume QUARC change | -0.210*** | -0.225*** | -0.255*** | -0.025 | 0.028 | -0.085 | 0.053 | 0.150* | -0.008 | 0.061 |
| Hippocampal volume QUARC change | -0.067 | -0.023 | 0.061 | 0.135 | -0.038 | 0.059 | 0.087 | 0.125 | -0.073 | 0.026 |
| Bilateral Lateral Ventricles volume QUARC change | 0.098 | 0.205** | 0.171* | 0.064 | 0.006 | 0.167* | 0.118 | -0.068 | 0.021 | -0.132 |
| Precuneus volume QUARC change | 0.007 | -0.040 | -0.024 | 0.071 | -0.001 | -0.008 | 0.040 | 0.042 | -0.073 | 0.035 |
| Isthmus volume QUARC change | -0.056 | -0.023 | -0.030 | 0.084 | 0.011 | -0.001 | 0.024 | 0.034 | -0.156* | 0.001 |
| Supramarginal volume QUARC change | -0.078 | -0.075 | -0.080 | 0.090 | 0.014 | -0.044 | 0.100 | 0.107 | -0.040 | 0.111 |

This table corresponds to the right heatmap in Figure 1b. * indicates p<0.05, ** indicates p<0.01, *** indicates p<0.001.

**Supplemental Table 3** Results of linear regression models predicting 48-week QUARC change for ROIs not significantly predicted by baseline plasma biomarker concentrations after adjusting for age, sex, years of education, APOE ε4 status, treatment arm, and baseline ADAS-Cog11 score.

|  | Std. coefficient | SE | t value | P value | 95% CI Lower.bound | 95% CI Upper.bound | Bonferroni adjusted P value |
| --- | --- | --- | --- | --- | --- | --- | --- |
| ***Entorhinal volume 48-week QUARC change*** | | | | | | | |
| GFAP | -0.17 | 0.15 | -1.08 | 0.283 | -0.47 | 0.14 | 1.000 |
| NfL | -0.19 | 0.15 | -1.21 | 0.229 | -0.49 | 0.12 | 1.000 |
| P-tau181 | -0.23 | 0.15 | -1.60 | 0.113 | -0.52 | 0.06 | 0.563 |
| T-tau | -0.08 | 0.14 | -0.53 | 0.600 | -0.35 | 0.21 | 1.000 |
| Aβ42/40 | -0.07 | 0.15 | -0.49 | 0.622 | -0.37 | 0.22 | 1.000 |
| ***Hippocampal volume 48-week QUARC change*** | | | | | | | |
| GFAP | -0.18 | 0.14 | -1.29 | 0.198 | -0.47 | 0.10 | 0.992 |
| NfL | -0.04 | 0.15 | -0.24 | 0.811 | -0.32 | 0.25 | 1.000 |
| P-tau181 | 0.20 | 0.14 | 1.48 | 0.141 | -0.07 | 0.47 | 0.707 |
| T-tau | 0.24 | 0.13 | 1.83 | 0.069 | -0.02 | 0.50 | 0.345 |
| Aβ42/40 | -0.02 | 0.14 | -0.16 | 0.871 | -0.30 | 0.25 | 1.000 |
| ***Whole Brain volume 48-week QUARC change*** | | | | | | | |
| GFAP | -0.28 | 0.16 | -1.73 | 0.085 | -0.60 | 0.04 | 0.426 |
| NfL | -0.24 | 0.16 | -1.44 | 0.152 | -0.56 | 0.09 | 0.759 |
| P-tau181 | -0.23 | 0.16 | -1.46 | 0.147 | -0.54 | 0.08 | 0.733 |
| T-tau | -0.20 | 0.15 | -1.32 | 0.190 | -0.49 | 0.10 | 0.951 |
| Aβ42/40 | -0.10 | 0.16 | -0.65 | 0.514 | -0.42 | 0.21 | 1.000 |
| ***Precuneus volume 48-week QUARC change*** | | | | | | | |
| GFAP | -0.04 | 0.12 | -0.34 | 0.733 | -0.28 | 0.20 | 1.000 |
| NfL | -0.11 | 0.12 | -0.88 | 0.383 | -0.35 | 0.14 | 1.000 |
| P-tau181 | -0.03 | 0.12 | -0.21 | 0.830 | -0.26 | 0.21 | 1.000 |
| T-tau | 0.18 | 0.11 | 1.57 | 0.117 | -0.05 | 0.40 | 0.586 |
| Aβ42/40 | 0.02 | 0.11 | 0.15 | 0.884 | -0.21 | 0.24 | 1.000 |
| ***Isthmus volume 48-week QUARC change*** | | | | | | | |
| GFAP | -0.10 | 0.11 | -0.89 | 0.373 | -0.32 | 0.12 | 1.000 |
| NfL | -0.03 | 0.11 | -0.22 | 0.824 | -0.25 | 0.20 | 1.000 |
| P-tau181 | 0.03 | 0.11 | 0.30 | 0.762 | -0.18 | 0.24 | 1.000 |
| T-tau | 0.15 | 0.10 | 1.50 | 0.134 | -0.05 | 0.36 | 0.670 |
| Aβ42/40 | 0.04 | 0.10 | 0.35 | 0.730 | -0.17 | 0.24 | 1.000 |
| ***Supramarginal volume 48-week QUARC change*** | | | | | | | |
| GFAP | -0.28 | 0.15 | -1.91 | 0.058 | -0.57 | 0.01 | 0.288 |
| NfL | -0.23 | 0.15 | -1.49 | 0.137 | -0.52 | 0.07 | 0.687 |
| P-tau181 | -0.14 | 0.14 | -0.98 | 0.326 | -0.41 | 0.14 | 1.000 |
| T-tau^*^ | 0.29 | 0.14 | 2.14 | 0.034 | 0.02 | 0.55 | 0.169 |
| Aβ42/40 | 0.12 | 0.14 | 0.91 | 0.365 | -0.14 | 0.39 | 1.000 |

**Supplemental Table 4** Comparing adjusted R-squared among LASSO selected model, combinations of LASSO selected biomarkers, and univariate biomarker models.

| 1. Linear regression model predicting ***48-week change in ADAS-Cog*** | Adjusted  R-squared |
| --- | --- |
| Age + Sex + Education + APOE + arm + Baseline ADAS-Cog score (naïve model) | 5.1% |
| naïve model + ***NfL*** | 6.8% |
| naïve model + ***T-tau*** | 4.4% |
| naïve model + ***Aβ42/40*** | 4.4% |
| naïve model + ***GFAP*** | 4.8% |
| naïve model + ***P-tau181*** | 4.5% |
| naïve model + ***T-tau + Aβ42/40*** | 4.3% |
| naïve model + ***T-tau + NfL*** | 7.3% |
| naïve model + ***Aβ42/40 + NfL*** | 6.8% |
| naïve model + ***T-tau + Aβ42/40 + NfL*** (LASSO selected model) | 7.4% |

| 1. Linear regression model predicting ***48-week change in CDR-SB*** | Adjusted  R-squared |
| --- | --- |
| Age + Sex + Education + APOE + arm + Baseline CDR-SB score (naïve model) | 3.6% |
| naïve model + ***NfL*** (LASSO selected model) | 5.4% |
| naïve model + ***T-tau*** | 3.8% |
| naïve model + ***Aβ42/40*** | 3.3% |
| naïve model + ***GFAP*** | 4.9% |
| naïve model + ***P-tau181*** | 4.2% |

**Supplemental Table 5** Multivariable LASSO regression selected models predicting 48-week change in ADAS-Cog11 (5A) or CDR-SB score (5B). Potential predictors included baseline plasma biomarkers and MRI volumes. Age, sex, education, APOE, arm assignment, and baseline score were forced into the LASSO models without penalty (naïve model).

| (5A) Linear regression model predicting ***48-week change in ADAS-Cog,*** n = 192 | | | | |
| --- | --- | --- | --- | --- |
| Predictors | Regression coefficients | Standard errors | t values | p values |
| Age | -0.27 | 0.07 | -3.71 | < 0.001 |
| Sex | 1.49 | 1.02 | 1.46 | 0.145 |
| Education | 0.28 | 0.17 | 1.67 | 0.096 |
| APOE ε4 carrier | 0.53 | 1.10 | 0.48 | 0.630 |
| Arm assignment | -0.84 | 0.98 | -0.86 | 0.392 |
| Baseline ADAS-Cog score | -0.02 | 0.06 | -0.27 | 0.790 |
| Baseline plasma NfL concentration | 0.07 | 0.05 | 1.50 | 0.135 |
| Baseline whole brain volume | -0.04 | 0.01 | -2.74 | 0.007 |
| Baseline precuneus volume | -0.55 | 0.49 | -1.13 | 0.262 |
| Adjusted R-squared increased from 5.1% (naïve model*) to 13.5% | | | | |
|  | | | | |
| (5B) Linear regression model predicting ***48-week change in CDR-SB,*** n = 192 | | | | |
| Predictors | Regression coefficients | Standard errors | t values | p values |
| Age | -0.04 | 0.03 | -1.44 | 0.152 |
| Sex | 0.20 | 0.36 | 0.55 | 0.580 |
| Education | 0.10 | 0.06 | 1.79 | 0.074 |
| APOE ε4 carrier | 0.29 | 0.37 | 0.78 | 0.439 |
| Arm assignment | -0.52 | 0.33 | -1.55 | 0.124 |
| Baseline CDR-SB score | -0.20 | 0.07 | -2.72 | 0.007 |
| Baseline plasma NfL concentration | 0.02 | 0.02 | 1.08 | 0.283 |
| Baseline ventricle volume | 0.01 | 0.02 | 0.52 | 0.605 |
| Baseline whole brain volume | -0.01 | 0.00 | -1.74 | 0.084 |
| Baseline precuneus volume | -0.12 | 0.19 | -0.62 | 0.535 |
| Baseline isthmus volume | -0.49 | 0.65 | -0.75 | 0.453 |
| Baseline mid-temporal volume | -0.13 | 0.13 | -0.99 | 0.326 |
| Baseline supramarginal volume | -0.16 | 0.23 | -0.70 | 0.484 |
| Adjusted R-squared increased from 3.6% (naïve model*) to 10.8% | | | | |

**Supplemental Table 6** Linear regression models testing whether 48-week change in each plasma biomarker was associated with 48-week change in ADAS-Cog11 (6A) or CDR-SB (6B) score.

|  | | (A) ADAS-Cog change | | | | | | |
| --- | --- | --- | --- | --- | --- | --- | --- | --- |
| Plasma biomarker | Standard coefficient | | SE | t value | P value | 95% CI Lower.bound | 95% CI Upper.bound | Bonferroni adjusted P value |
| GFAP | 0.48 | | 0.52 | 0.92 | 0.357 | -0.54 | 1.50 | 1.000 |
| NfL | -0.07 | | 0.50 | -0.14 | 0.890 | -1.06 | 0.92 | 1.000 |
| P-tau181 | -0.51 | | 0.51 | -1.01 | 0.316 | -1.50 | 0.49 | 1.000 |
| T-tau | 0.19 | | 0.49 | 0.38 | 0.701 | -0.78 | 1.16 | 1.000 |
| Aβ42/40 | 0.65 | | 0.50 | 1.30 | 0.194 | -0.33 | 1.63 | 0.969 |
|  | | (B) CDR-SB change | | | | | | |
| Plasma biomarker | Standard coefficient | | SE | t value | P value | 95% CI Lower.bound | 95% CI Upper.bound | Bonferroni adjusted P value |
| GFAP | 0.31 | | 0.16 | 1.92 | 0.057 | -0.01 | 0.63 | 0.284 |
| NfL | 0.43 | | 0.16 | 2.75 | 0.007 | 0.12 | 0.74 | 0.033 |
| P-tau181 | -0.27 | | 0.16 | -1.68 | 0.095 | -0.59 | 0.05 | 0.473 |
| T-tau | 0.14 | | 0.16 | 0.92 | 0.356 | -0.16 | 0.45 | 1.000 |
| Aβ42/40 | 0.12 | | 0.16 | 0.74 | 0.460 | -0.20 | 0.43 | 1.000 |

**Supplemental Figure 1**


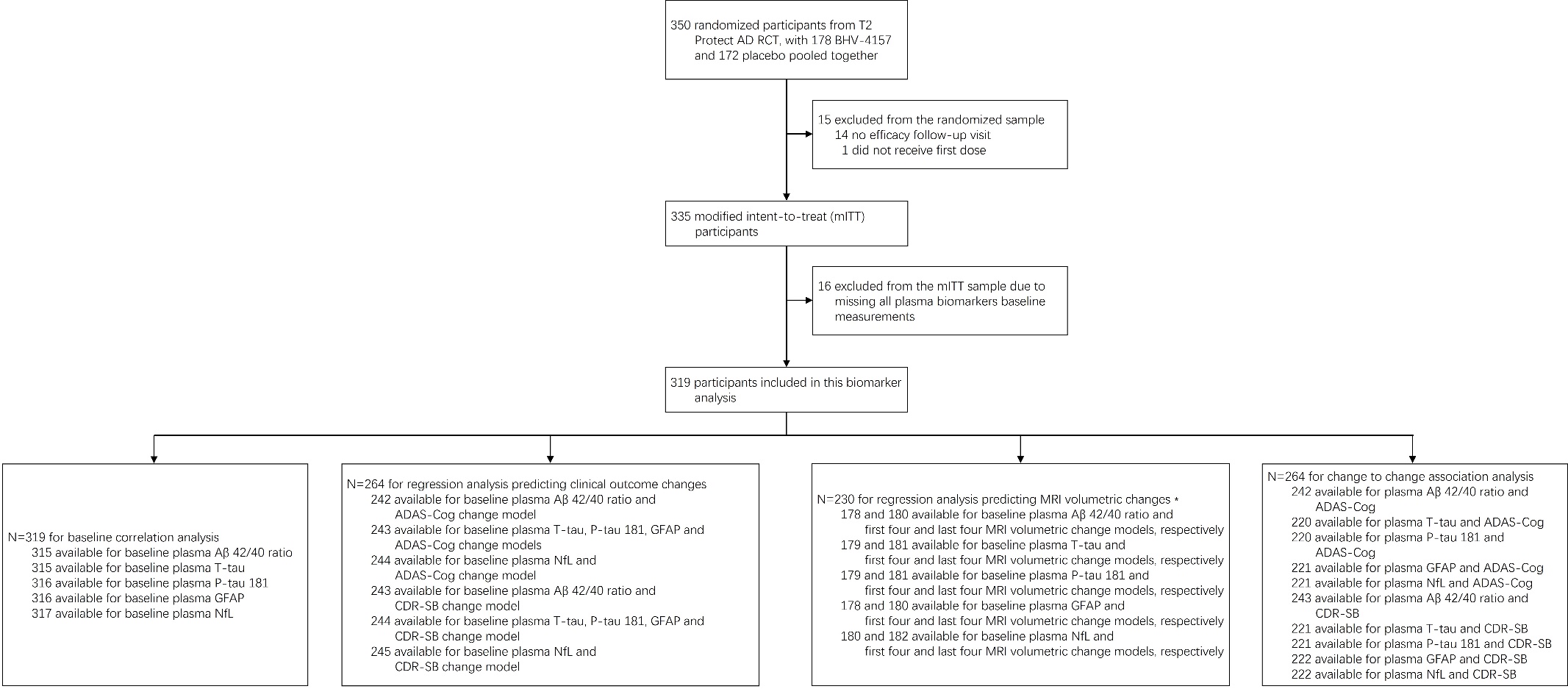


*The MRI volumetric measures are in the following order: entorhinal, hippocampal, bilateral lateral ventricles, whole brain, precuneus, isthmus, mid-temporal, and supramarginal volumes.

**Supplemental Figure 2**


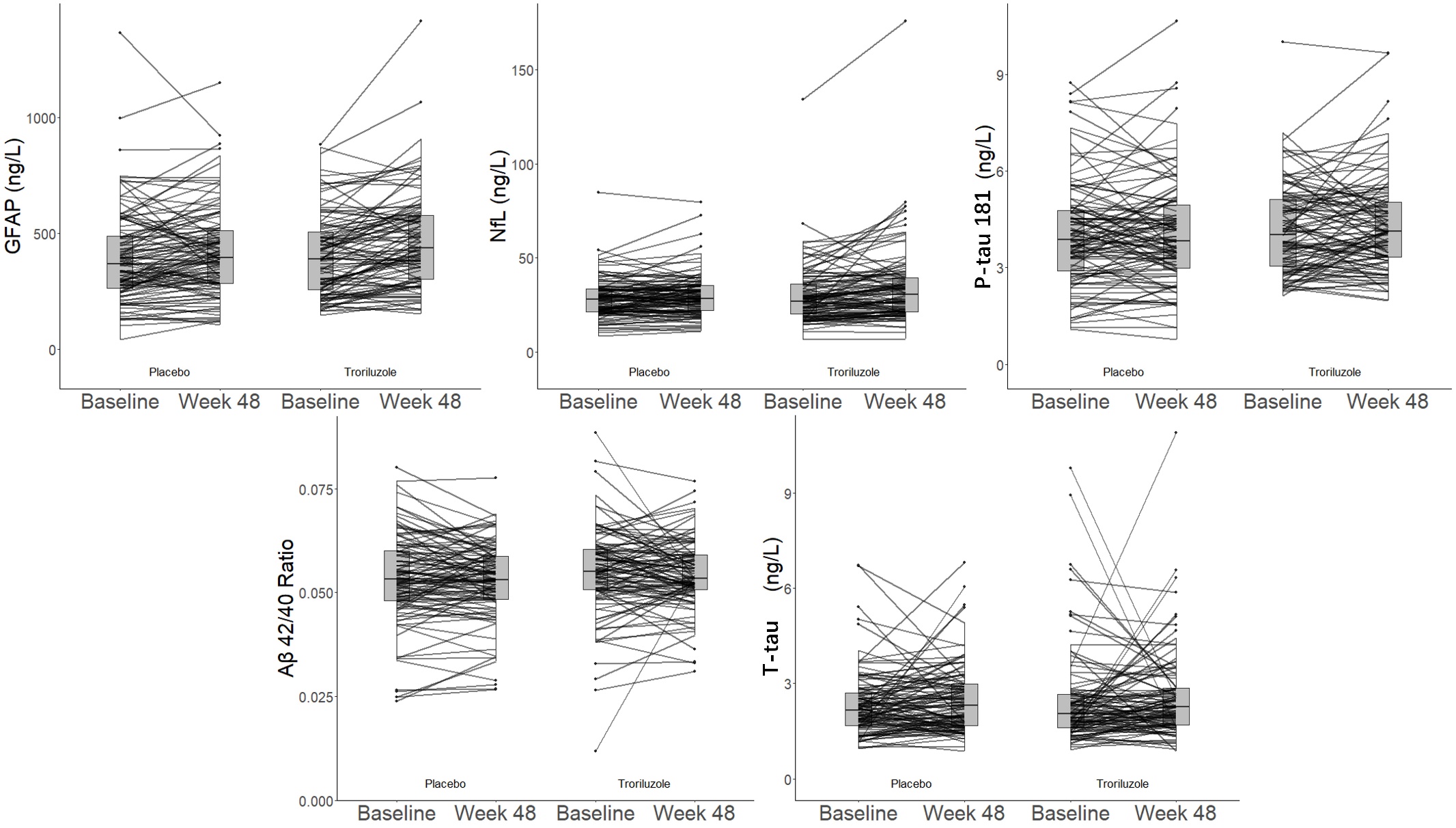


One patient in the placebo group with an unusual concentration of baseline T-tau (36.6 ng/L) was identified as an outlier and removed from the T-tau boxplots.

**Supplemental Figure 3**

(a)


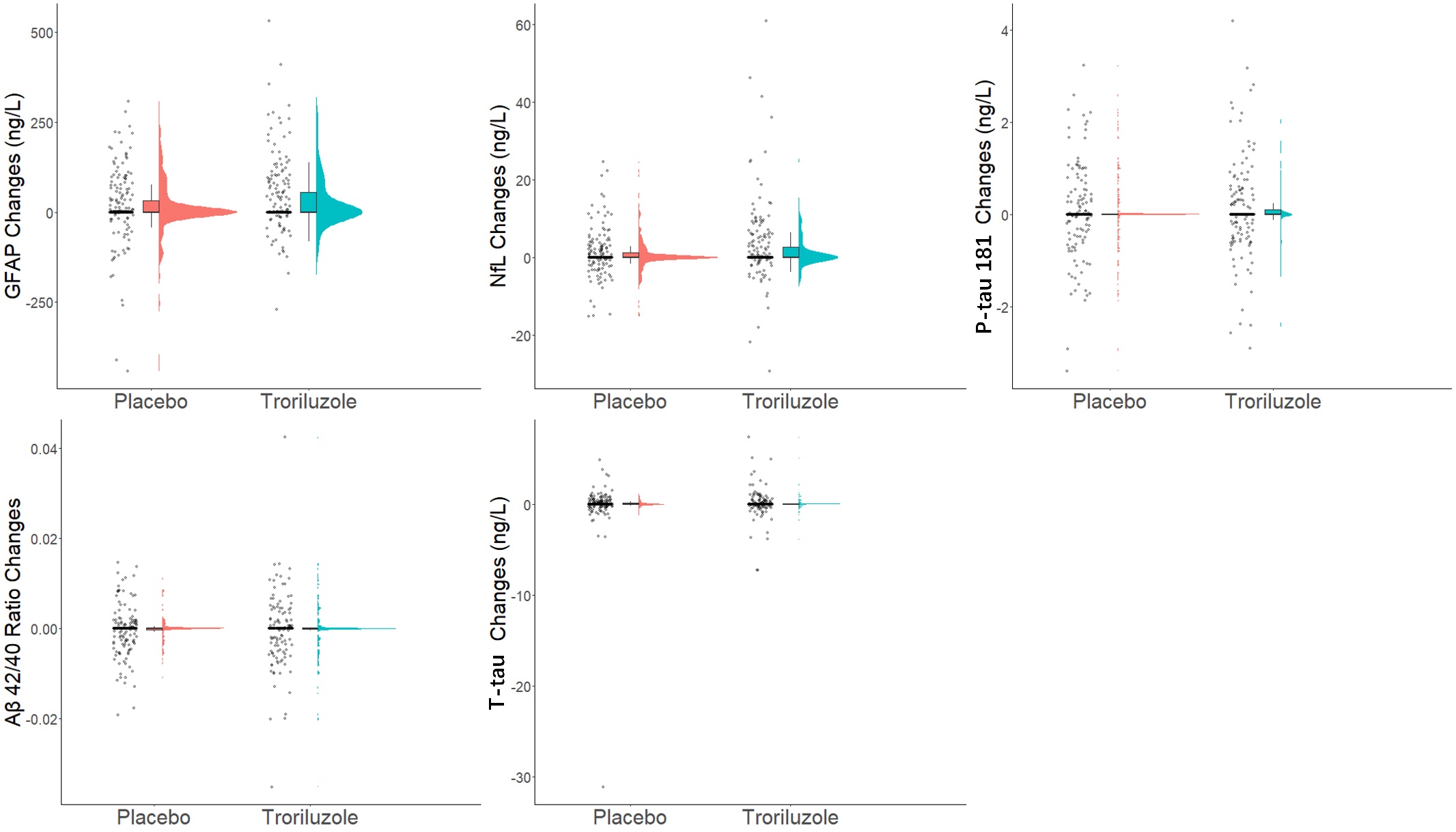


(b)


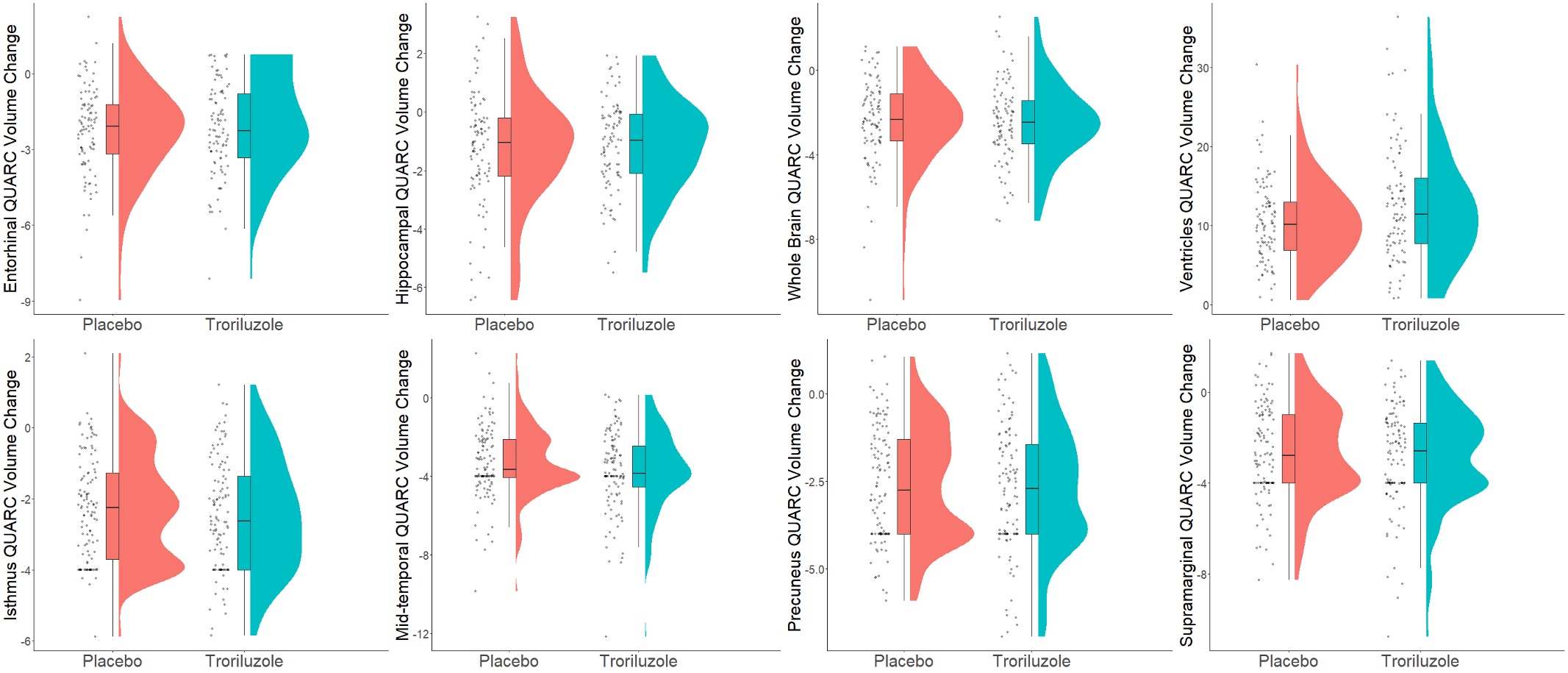

Supplement: Supplementary file 1 — Supplementary Material 1. [file 13195_2025_1745_MOESM1_ESM.docx]
